# From Unit to Dose: A Machine Learning Approach for Precise Prediction of Hemoglobin and Iron Content in Individual Packed Red Blood Cell Units

**DOI:** 10.1002/advs.202204077

**Published:** 2022-11-04

**Authors:** Jeremy Epah, Ilay Gülec, Stefan Winter, Johanna Dörr, Christof Geisen, Eva Haecker, Dietmar Link, Matthias Schwab, Erhard Seifried, Richard Schäfer

**Affiliations:** ^1^ Institute for Transfusion Medicine and Immunohaematology, German Red Cross Blood Donor Service Baden‐Württemberg‐Hessen gGmbH Goethe University Hospital 60528 Frankfurt am Main Germany; ^2^ Dr. Margarete Fischer‐Bosch Institute of Clinical Pharmacology Stuttgart, Germany University of Tübingen 72076 Tübingen Germany; ^3^ Departments of Clinical Pharmacology Pharmacy and Biochemistry University of Tübingen 72076 Tübingen Germany; ^4^ Cluster of Excellence iFIT (EXC 2180), Image‐Guided and Functionally Instructed Tumor Therapies“ University of Tübingen 72076 Tübingen Germany; ^5^ Institute for Transfusion Medicine and Gene Therapy Medical Center – University of Freiburg 79106 Freiburg Germany; ^6^ Center for Chronic Immunodeficiency (CCI) Medical Center – University of Freiburg 79106 Freiburg Germany

**Keywords:** hemoglobin, iron, machine learning, packed red blood cells, personalized medicine, prediction, transfusion

## Abstract

Transfusion of packed red blood cells (pRBCs) saves lives, but iron overload limits survival of chronically transfused patients. Quality control methods, which involve entering pRBC units and removing them from the blood supply, reveal that hemoglobin (38.5–79.9 g) and heme iron (133.42–276.89 mg) vary substantially between pRBCs. Yet, neither hemoglobin nor iron content can be quantified for individual clinically used pRBCs leading to rules of thumb for pRBC transfusions. Keeping their integrity, the authors seek to predict hemoglobin/iron content of any given pRBC unit applying eight machine learning models on 6,058 pRBCs. Based on thirteen features routinely collected during blood donation, production and quality control testing, the model with best trade‐off between performance and complexity in hemoglobin/iron content prediction is identified. Validation of this model in an independent cohort of 2637 pRBCs confirms an adjusted *R* ^2^ > 0.9 corresponding to a mean absolute prediction error of ≤1.43 g hemoglobin/4.96 mg iron (associated standard deviation: ≤1.13 g hemoglobin/3.92 mg iron). Such unprecedented precise prediction enables reliable pRBC dosing per pharmaceutically active agent, and monitoring iron uptake in patients and individual iron loss in donors. The model is implemented in a free open source web application to facilitate clinical application.

## Graphical abstract

Hemoglobin and iron content vary substantially between packed red blood cell (pRBC) units, leading to rules of thumb for blood transfusions. Machine learning now allows to precisely predict hemoglobin and iron content of individual pRBC units, enabling for the first time reliable pRBC dosing per pharmaceutically active agent, and monitoring iron uptake in patients and individual iron loss in donors.

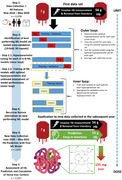

## 1 Introduction

The World Health Organization has acknowledged that blood transfusions are lifesaving interventions with an essential role in patient management.^[^ ^1^ ^]^ Thus, providing hemoglobin (Hb) as oxygen carrier to prevent manifest anemia‐related tissue hypoxia, transfusion of packed red blood cells (pRBCs) is implemented in therapy regimes of acute or chronic anemia.^[^ ^2^, ^3^ ^]^ However, chronic Hb delivery comes with substantial side effects. Inducing reactive oxygen species, such as hydroxyl radicals and lipid peroxidative products, non‐transferrin‐bound iron (NTBI) causes damages in various organs.^[^ ^4^ ^]^ Subsequently, while saved from chronic hypoxemia by provision of oxygen carrying Hb, patients depending on regular blood transfusions face the fatal risk of chronic iron overload^[^ ^4^ ^]^ that is associated with a decreased overall survival in myelodysplastic patients and is associated with a higher frequency of early and late complications in hematopoietic stem cell transplantation.^[^ ^5^ ^]^ Notably, 20% of children with *β*‐thalassemia major die by the age of 15 with refractory myocardial failure due to iron overload as major cause of death, and 40% of young *β*‐thalassemia patients die by the age of 20 if not treated with costly and side effect prone chelation therapy.^[^ ^6^, ^7^, ^8^ ^]^

Yet, precise iron monitoring remains difficult as measurement of serum ferritin cannot determine iron balance directly, does not follow high iron load levels linearly, and is affected by inflammation. Determination of liver iron content requires costly magnetic resonance imaging with limited availability, and liver biopsies come with risks of infections and bleeding.^[^ ^9^ ^]^

Despite the fact that pRBCs vary clearly in their total Hb content and, accordingly their heme iron content, with a range up to 100%,^[^ ^10^ ^]^ to date neither concentrations of the pharmaceutically active agent (i.e., Hb) nor of the potential toxic agent (i.e., heme iron) are known for individual pRBC units and thus, cannot be considered for transfusions. This has led to the fact that rules of thumb have been applied for clinical pRBC transfusions worldwide. For example, in pediatrics it is assumed that 5 mL pRBC unit/kg body weight will raise Hb concentration in patient's blood for ≈1 g dL^−1^,^[^ ^11^ ^]^ and in adults a similar result would be achieved by transfusing one complete pRBC unit.^[^ ^12^ ^]^ Thus, in context of transfusion‐associated chronic iron overload, considering patient blood management (PBM) programs and changing demographics with dramatic impact on blood supply,^[^ ^13^, ^14^ ^]^ a more rational and evidence‐based use of pRBCs is imperative. In fact, it was previously shown that Hb‐based dosing can effectively reduce pRBC usage in the clinic.^[^ ^15^ ^]^ Importantly, heme iron content in pRBCs does not only reflect iron being transfused to patients, but also the donor's individual iron loss. Pre‐donation Hb testing only detects donors with manifest iron deficiency anemia, but is poorly sensitive when it comes to pre‐anemic iron deficiency.^[^ ^16^ ^]^ Thus, accurately determining the individual Hb and heme iron loss per donation is expected to improve blood donors’ safety.

To date, quality control testing of pRBCs' Hb content can only be performed for a minute percentage of the pRBC production with these products being discharged and such removed from the inventory. Previous attempts to find the “best fit” pRBC unit from the inventory for a given patient were carried out by estimating total Hb content of a pRBC unit simply by multiplying the volume of the whole blood donation with the measured donor's fingertip Hb value.^[^ ^17^, ^18^ ^]^ A related approach additionally subtracts the measured blood volume lost due to leucofiltration,^[^ ^10^ ^]^ but this only slightly affects the predicted Hb content. Importantly, a validation of these approaches with real Hb measurements was not reported,^[^ ^10^, ^15^, ^17^, ^18^ ^]^ which is yet an essential prerequisite to assess clinical applicability. Thus, we first tested these previously published concepts in a pilot study, where it became clear that, due to their severely limited performance, a more accurate concept was required. This prompted us to develop and establish a machine learning (ML) model for the prediction of Hb and iron content in individual pRBC units based on features routinely collected during blood donation, production, and quality control testing.

ML is revolutionizing medicine increasingly, supporting medical professionals in decision making, for example, in classifying skin cancer images^[^ ^19^ ^]^ or predicting the progression from pre‐diabetes to type 2 diabetes.^[^ ^20^ ^]^

Regarding the problem of precise Hb and iron content prediction in individual pRBC units, we hypothesized that a multi‐feature approach based on the parameters being routinely recorded during blood donation and pRBC production could outperform previous attempts. We therefore tested if supervised ML approaches could provide reliable predictions of Hb and iron content in pRBC units. Since the functional form of the relationship between Hb and iron content and independent parameters was completely unknown, we started with an unbiased approach and thoroughly tested various different ML methods. Specifically, we trained eight different ML algorithms on donation and quality control data of 6058 pRBC units that were collected between May 2018 and May 2020 and manufactured at five different production sites. Performance of the finally selected model was assessed in a second, independent data set of 2637 pRBCs of the subsequent year. Herein, we verified our hypothesis that a supervised ML approach can deliver a non‐invasive, automated method to predict the Hb and iron content in individual pRBC units with high precision.

## 2 Experimental Section

### 2.1 Production of pRBCs

The pRBC units were produced from whole blood donations varying between 450 to 515 mL collected from male and female European citizens ranging in age from 18 to 73 years, with pre‐donation fingertip Hb‐levels from 12.5–16.5 g dL^−1^ for women and 13.5–18.5 g dL^−1^ for men. The whole blood donations were collected with the CompoFlow blood bag system CQ42271 by Fresenius Kabi Deutschland GmbH, Bad Homburg vor der Höhe, Germany, or with the blood bag system LQT7248LC by Maco Pharma Int. GmbH, Langen, Germany, and processed with the CompoMat G5 Plus by Fresenius Kabi.

### 2.2 Software

For ML and data visualization tasks the open source ecosystem and data science tool kit Anaconda and its application Jupiter Notebook 6.0.3,^[^ ^21^ ^]^ Python Version 3.8,^[^ ^22^ ^]^ and Google Colaboratory^[^ ^23^ ^]^ was used. Applied programming libraries were Pandas 1.2.0.,^[^ ^24^ ^]^ Numpy 1.18.5,^[^ ^25^ ^]^ Matplotlib 3.2.2,^[^ ^26^ ^]^ Scikit learn 0.23.1,^[^ ^27^ ^]^ Seaborn 0.10.1,^[^ ^28^ ^]^ Scipy 1.5.0,^[^ ^29^ ^]^ pyCompare 1.5.3,^[^ ^30^ ^]^ and TensorFlow^[^ ^31^ ^]^/Keras 2.3.1. To use Keras modules within the Scikit learn framework the wrapper scikeras 0.2.1^[^ ^32^ ^]^ was used. For statistical analysis the software Graph Pad Prism 5 (GraphPad Software, San Diego, CA, USA) was used. The 3D structure of the hemoglobin molecule and the chemical structure of the heme group in the ToC figure were created with BioRender.com.

### 2.3 Data Collection

As per regulatory obligation one percent of the monthly pRBC production was tested for quality control. To obtain representative samples from these pRBC units for analysis they need to be opened and, thus, removed from the inventory. The use of data for scientific purposes was approved by the Institutional Review Board of the University Hospital Frankfurt, Germany (Approval# 329/10). The cohorts are described in **Table** **1**. For ML model development a data set was employed that comprised all parameters (except for individual donor questionnaire) being routinely collected during manufacturing and quality control of randomly picked 6058 pRBCs produced at five different production sites (including international metropolitan agglomerations and rural areas) by the German Red Cross Blood Donor Service Baden‐Württemberg Hessen in the period of May 2018 to May 2020 (Table 1). In detail, the following routinely collected features *n* = 13 were accessible from the authors’ blood banking system: total Hb content in grams, hematocrit (Hct) in percent, total unit volume in milliliters, extracted plasma volume in milliliters, production site, production date, donor age, donor sex, donor Hb in grams per deciliter obtained from obligatory pre‐collection fingertip testing, the volume of the whole blood collection in milliliters, type of blood bag system, production machine, and program (Table 1). A second, independent data cohort comprising 2637 units collected in the period of July 2020 to May 2021 was used to assess the performance of the final model (Table 1). The target value total Hb content in grams per unit followed well a normal distribution in both data sets (Figure S1, Supporting Information).

**Table 1:** Description of first and second, independent data set

**First data set**	***n***	**mean**	**SD**	**min**	**25%**	**50%**	**75%**	**max**
Unit volume [mL]	6058	293.19	18.95	230	279.25	293	307	369
Unit Hb [g/unit]	6058	56.21	6.35	38.5	51.6	56.1	60.8	79.9
Fingertip Hb [g dL^−1^]	6058	14.95	1.29	11.4	14	14.9	15.9	18.9
Separated plasma volume [mL]	6058	314.72	23.24	225	299	315	331	672
Whole blood donation volume [mL]	6058	499.71	4.52	450	500	500	501	514
Donor age [years]	6058	44.04	15.04	18.00	30.39	46.17	55.89	72.80

**Second data set**	***n***	**mean**	**SD**	**min**	**25%**	**50%**	**75%**	**max**
Unit volume [mL]	2637	291.69	18.41	215.5	278.5	291.7	304.6	349.8
Unit Hb [g/unit]	2637	55.80	6.24	38	51.3	55.8	60.3	77.7
Fingertip Hb [g dL^−1^]	2637	14.97	1.31	11.9	14	14.9	15.9	18.5
Separated plasma volume [mL]	2637	313.49	22.62	211	298	314	330	378
Whole blood donation volume [mL]	2637	499.73	4.90	450	500	500	501	528
Donor age [years]	2637	44.53	15.07	18.01	30.86	47.26	56.37	72.97

**First data set**		**Second data set**	
**Production site**	***n***	**Production site**	***n***
Site_A	1036	Site_A	227
Site_B	1043	Site_B	158
Site_C	1027	Site_C	808
Site_D	2158	Site_D	864
Site_E	794	Site_E	580

**Month**		**Month**	
April	386	April	233
August	435	August	186
December	492	December	118
February	658	February	260
January	923	January	88
July	424	July	212
June	276	June	71
March	731	March	300
May	484	May	186
November	496	November	341
October	388	October	252
September	365	September	390

**Sex**		**Sex**	
Female	2546	Female	1191
Male	3512	Male	1446

**Blood Group**		**Blood Group**	
A	424	A	64
AB	1754	AB	870
B	3831	B	1683
O	49	O	19
Rhesus		Rhesus	
Rh neg	28	Rh neg	9
Rh pos	6030	Rh pos	2628

**Blood bag system**		**Blood bag system**	
CQ42271 FRESENIUS KABI DEUTSCHLAND GMBH	4773	CQ42271 FRESENIUS KABI DEUTSCHLAND GMBH	2279
LQT7248LC MACO PHARMA INT. GMBH	1,285	LQT7248LC MACO PHARMA INT. GMBH	358

**Machine**		**Machine**	
702COA	84	702COA	6
702COB	75	702COB	10
702COC	77	702COC	6
702COD	69	702COD	7
702COE	82	702COE	8
702COF	73	702COF	12
702COG	102	702COG	6
702COH	86	702COH	10
702COI	80	702COI	11
702COJ	86	702COJ	14
702COK	83	702COK	12
702COL	81	702COL	11
702COM	82	702COM	15
702CON	88	702CON	5
702COO	73	702COO	12
702COP	73	702COP	7
702COQ	74	702COQ	8
702COR	80	702COR	12
702COS	68	702COS	2
702COT	83	702COT	8
702COU	86	702COU	10
702COV	93	702COV	12
702COW	71	702COW	16
702COX	73	702COX	14
704COA	6	704COE	167
704COB	5	704COF	149
704COC	6	704COG	160
704COD	7	704COH	154
704COE	268	704COI	159
704COF	143	704COJ	150
704COG	213	704COK	142
704COH	243	704COL	152
704COI	184	704COM	132
704COJ	246	704CON	146
704COK	225	704COO	101
704COL	262	704COP	91
704COM	230	704COQ	82
704CON	223	704COR	79
704COO	181	704COS	70
704COP	224	704COT	150
704COQ	182	704COU	160
704COR	180	704COV	158
704COS	211		
704COT	275		
704COU	197		
704COV	260		
704COW	48		
704COX	13		
704COY	51		
704COZ	53		

**Program**		**Program**	
Eight	504	Eight	63
Fifteen	41	Five	5
Five	450	Four	808
Four	1361	Nineteen	243
Nineteen	367	Six	166
One	62	Three	1242
Seven	117	Twenty	110
Seventeen	3		
Six	815		
Three	1997		
Twenty	307		
Two	34		

### 2.4 Training and Evaluation of ML Algorithms

First, categorical variables such as sex and month of production were transformed into integer variables using One Hot Coding.^[^ ^33^ ^]^ Eight common ML algorithms including multiple linear regression (MLR), random sample consensus (RANSAC)^[^ ^34^ ^]^ algorithm, support vector machine (SVM),^[^ ^35^ ^]^ Random Forest (RF),^[^ ^36^ ^]^ k‐Nearest Neighbor (KNN),^[^ ^37^ ^]^ decision tree (DecTree),^[^ ^38^ ^]^ light GBM Regressor (lgbmR),^[^ ^39^ ^]^ and neural networks (NN)^[^ ^40^ ^]^ were evaluated on the first data set to determine the best performing ML model using 50‐times repeated nested tenfold cross‐validation in the Scikit learn library^[^ ^27^ ^]^ (outer and inner loop, graphical abstract) and all 13 routinely collected features. Here, GridSearchCV was applied for hyper‐parameter tuning of KNN (n_neighbors, weights, algorithm, leaf_size), SVM (C, epsilon, gamma, kernel), DecTree (min_samples_split, min_samples_leaf, splitter), RF (max_features, n_estimators) and NN (activation, optimizer, number of neurons, size of layers, batch size, epochs, optimizer, kernel initializer, dropout rate) (inner loop, graphical abstract). Throughout this article, the mean squared error (MSE) is used as loss function. In addition, for easier interpretation of results, the closely related coefficient of determination (*R* ^2^) was reported, which can be regarded as rescaled or normalized version of the MSE.

For the best performing ML model in terms of lowest median MSE on the first data set—in this case MLR—feature selection was conducted subsequently. Here, feature ranking with recursive feature elimination and cross‐validated selection of the best number of features (RFECV) from the Scikit learn library was applied using default settings, a hyper‐parameter tuned DecTree Regressor (see above) as estimator and 50‐times repeated nested tenfold cross‐validation. Model performance of MLR with the best combination of all, three or four features, was then evaluated using 50‐times repeated tenfold cross‐validation. The two models with the best trade‐off between performance and complexity—MLR with 3 and 4 features, respectively—were retrained on the entire first data set (*n* = 6058) and subsequently evaluated on the second, independent data set (*n* = 2637) for an unbiased assessment of model performance.

### 2.5 Calculation or Prediction of a pRBC Units' Heme Iron Content

Given a mean molecular weight of the Hb tetramer of 64.458 g mmol^−1[^ ^41^ ^]^ and the fact that one Hb tetramer contains four iron atoms with a molar mass of 55.845 g mol^−1^ applying the common rules of chemical stoichiometry, the following equation was utilized to calculate or predict heme iron content based on the measured or estimated Hb content in a pRBC unit:

(1) $$\mathrm{heme}\;\mathrm{iron}\left(\mathrm{mg}/\mathrm{unit}\right)=\frac{x*4\mathrm{Fe}*55.845\;g\;{\mathrm{mol}}^{-1}}{64.458\;g\;{\mathrm{mmol}}^{-1}}$$

*x* = measured or estimated Hb content in g Hb/unit.

### 2.6 Pilot Study for Hb Content Calculation in pRBC Units

pRBCs (*n* = 9) were randomly picked independent from the first as well as the second cohort. For these samples, the measured Hb content and the calculated Hb content were compared based on the equations of Arslan et al.^[^ ^15^ ^]^ and Agnihotri et al.^[^ ^10^ ^]^

After weighing and gently shaking on a rocker, samples from the pRBC units were taken in S‐Monovettes with potassium EDTA, 2.7 mL (Sarstedt AG & Co. KG, Nümbrecht, Germany). Hb concentration in g dL^−1^ and Hct in percentage were determined by two repeated measurements with the Sysmex XN‐350 System (Sysmex Deutschland GmbH, Norderstedt, Germany). Unit volume was calculated dividing the measured weight by the relative density of blood^[^ ^42^ ^]^ adjusted to the measured Hct in percentage. Total Hb content in the pRBC units was calculated by multiplying the measured Hb concentration with the respective unit volume.

### 2.7 Calculating the Hb Content in pRBC Units as Proposed by Arslan et al.^[^ ^15^ ^]^ and Atilla et al.^[^ ^18^ ^]^

(2) $$\begin{array}{ccc} & & \mathrm{Predicted}\;\mathrm{total}\;\mathrm{Hb}\;\mathrm{in}\;\mathrm{unit}\left(g\right)=\mathrm{Whole}\;\mathrm{blood}\;\mathrm{donation}\left(L\right)*\\ & & \;\mathrm{Fingertip}\;\mathrm{Hb}\left(\frac{g}{L}\right) \end{array}$$

Calculating the Hb content in pRBC units as proposed by Agnihotri et al:^[^ ^10^ ^]^

(3) $$\begin{array}{ccc} & & \mathrm{Predicted}\;\mathrm{total}\;\mathrm{Hb}\;\mathrm{in}\;\mathrm{unit}\left(g\right)=\mathrm{Whole}\;\mathrm{blood}\;\mathrm{donation}\left(L\right)*\\ & & \;\mathrm{Fingertip}\;\mathrm{Hb}\left(\frac{g}{L}\right)-0.035\left(L\right)*\mathrm{Fingertip}\;\mathrm{Hb}\left(\frac{g}{L}\right) \end{array}$$

Further information as well as the derivation of both equations can be found in the Supporting Information.

## 3 Data Availability

Due to data protection law individual donor data cannot be disclosed. De‐identified processed data that underlie the results reported in this article will be shared upon reasonable request after signing a data protection agreement and a data access agreement. Investigators requesting data must also provide a methodologically sound proposal describing the aims that should be achieved by using the data. Proposals should be directed to j.epah@blutspende.de for the above mentioned agreements, to submit their research proposal and to gain access to the processed data.

## 4 Code Availability

The additional customized code, besides the standard, open source data science and machine learning libraries, is available at https://github.com/epahjeremy/prbc‐prediction.

## 5 Results

### 5.1 Hemoglobin and Iron Content is Highly Variable in pRBC Units

**Figure 1:**
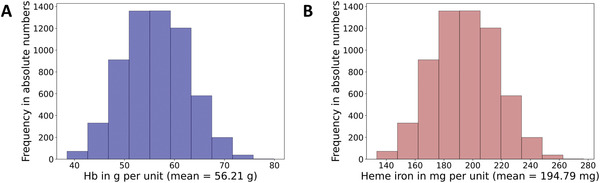
Histograms of measured Hb and calculated iron content in first data set (*n* = 6058). A) Histogram of measured Hb content in 6058 pRBC units. B) Histogram of calculated heme iron content in 6058 pRBC units.

When we analyzed our first data set of 6058 pRBC units we found a great variability of the Hb content ranging from 38.5 to 79.9 g per unit with a mean of 56.21 g Hb. We discovered that only 60% of all tested pRBC units contained 56.21g ± 10% of the mean (5.62 g) Hb, whereas 32% deviated greater than 10% from the mean and 8% even greater than 20% (**Figure** **1**A).

As shown Equation (1) heme iron represents total iron within a pRBC unit and is determined by the Hb content. Therefore, we calculated heme iron based on the respective Hb levels. According to the Hb distribution 60% of the pRBC units contained 194.74 ± 10% of the mean (19.47 mg) iron, whereas 32% deviated greater than 10% from the mean and 8% even greater than 20% (Figure 1B).

### 5.2 Previous Hb Content Prediction Approaches Substantially Overestimate Hb Content in pRBC Units

**Figure 2:**
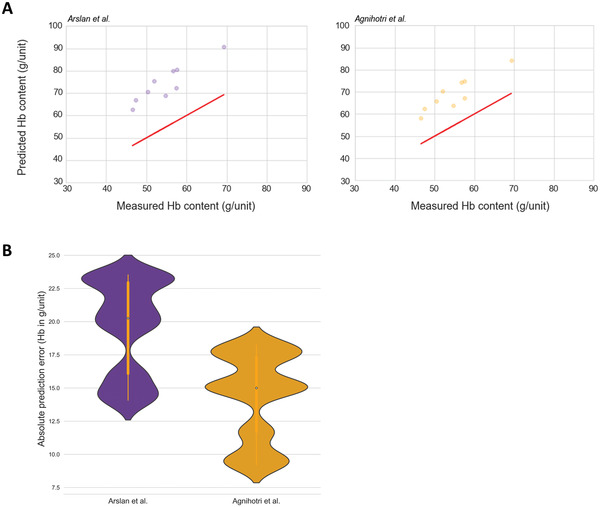
Comparison of Hb prediction approaches by Arslan et al., and Agnihotri et al. to measured Hb content in preliminary tested pRBC units (*n* = 9). A) Scatter plot of measured Hb content in g/unit (red) in the preliminary tested pRBC units versus predicted Hb content applying the equations of Arslan et al. (purple) (Equation (2)) and Agnihotri et al. (gold) (Equation (3)). B) Violin plots of absolute prediction errors generated by the equations of Arslan et al. (Equation (2)), and Agnihotri et al. (Equation (3)) on the preliminary tested pRBC units.

In a pilot study comprising 9 pRBC units, we used the previous approaches developed by Arslan et al.^[^ ^15^ ^]^ and Agnihotri et al.^[^ ^10^ ^]^ to estimate the Hb content. Of note, both approaches substantially overestimated the measured (real) Hb content with mean prediction errors of 19.55 g Hb/unit (30.8%) and 14.35 g Hb/unit (26.4%) (**Figure** **2**A,B).

### 5.3 Multiple Linear Regression, SVM, and LGBMR are Best Performing Machine Learning Models for Hb and Iron Prediction in pRBC Units

**Figure 3:**
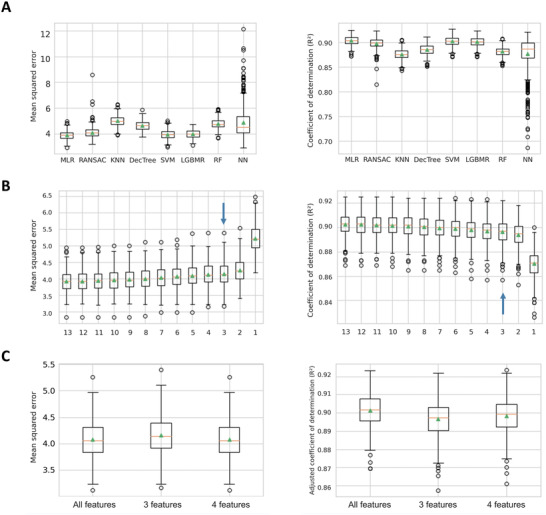
Identification of the ML model performing best on the first data set (*n* = 6058). A) Mean squared errors (MSE) and coefficients of determination (*R* ^2^) of eight different ML models for Hb prediction using all 13 features as input variables; 50 times repeated nested tenfold CV. MLR = Multiple Linear Regression; RANSAC = Random Sample Consensus; KNN = k‐nearest neighbor, DecTree = tree, SVM = Support Vector Machine; LGBMR = lightGBM Regressor; RF = Random Forest; NN = Neural network. Green triangles indicate means, orange lines indicate medians; upper and lower box borders indicate interquartile range (25.0–75.0 percentile); error bars: 99.7% CI. B) Mean squared errors (MSE) and coefficients of determination (*R* ^2^) of MLR models for Hb prediction with recursive feature elimination using between all to one feature as input variables; 50 times repeated nested tenfold CV. Blue arrow highlights best number of features by RFECV. Green triangles indicate means, orange lines indicate medians; upper and lower box borders indicate interquartile range (25.0 to 75.0 percentile); error bars: 99.7% CI. C) Mean squared errors (MSE) and adjusted coefficients of determination (*R* ^2^) of different MLR models for Hb prediction using all, 3 and 4 features as input variables; 50‐times repeated tenfold CV. Green triangles indicate means, orange lines indicate medians; upper and lower box borders indicate interquartile range (25.0–75.0 percentile); error bars: 99.7% CI.

As the previously developed approaches of Arslan et al.^[^ ^15^ ^]^ and Agnihotri et al.^[^ ^10^ ^]^ showed poor accuracy in our pilot study, we sought to develop our own model to predict Hb and iron content of any given pRBC unit without compromising the integrity of these products. Therefore, we considered all (thirteen) features that were routinely collected during the blood donation, pRBC production and quality control, and evaluated eight different ML methods on the first data set. Specifically, we found that MLR, RANSAC, SVM and LGBMR slightly outperformed DecTree, KNN, RF and NN with respect to median MSE and median *R* ^2^ on the first data set, with MLR performing the best (median MSE for Hb prediction = 3.89, 95% confidence interval = 3.3–4.5; median *R* ^2^ = 0.903, 95% confidence interval = 0.885–0.921) (**Figure** **3**A).

### 5.4 Recursive Feature Elimination Selects Unit Volume, Donor Hb Obtained by Fingertip Testing, and Donor Sex as the Best Feature Combination in MLR

Out of the best performing ML methods, we selected the MLR for its simplicity and easy applicability and used cross‐validation based feature section (RFECV) on the first data set (*n* = 6058) to identify the optimal number of features. This number turned out to be three with the best combination being unit volume, donor Hb obtained by fingertip testing, and donor sex (Figure 3B). Nevertheless, we observed that the model with four features performed almost as well with a slightly decreased mean and median cross‐validated MSE and slightly increased mean and median cross‐validated *R* ^2^ (Figure 3B). Here, the blood bag system was additionally part of the best combination. Interestingly, in each of the 50 cross‐validation repeats, the same features for the three as well as for the four‐feature model were selected. Both sets of features reached almost an as low MSE and as high adjusted *R* ^2^ as the MLR with all features (mean/median MSE for Hb prediction ≈4.1; mean/median *R* ^2^ ≈0.9), when applying the models on the entire first data set (Figure 3C). Since we supposed that the feature blood bag system may be relevant in the production process, we decided to consider both MLR with three and four features for an unbiased assessment of model performance in our second, independent data set (*n* = 2637).

Considering the above described iterative process, we propose the two following equations for Hb and iron prediction in pRBC units:

### 5.5 Equation for MLR with Three Features

(4) $$\begin{array}{ccc} & & \mathrm{Predicted}\;\mathrm{total}\;\mathrm{Hb}\;\mathrm{in}\;\mathrm{unit}\left(g\right)=\beta 0+\beta 1*\mathrm{Unit}\;\mathrm{volume}\left(\mathrm{mL}\right)\\ & & \;+\;\beta 2*\mathrm{Hb}\;\mathrm{Fingertip}\left(\frac{g}{\mathrm{dL}}\right)+\beta 3*\mathrm{xi}3+\beta 4*\mathrm{xi}4 \end{array}$$

*β*0 = (−32.771); *β*1 *=* 0.254; *β*2 *=* 0.964;

*β*3 = (‐0.426); *β*4 = 0.426

*xi*3 = 1 if female, else 0;

*xi*4 = 1 if male, else 0.

### 5.6 Equation for MLR with Four Features

(5) $$\begin{array}{ccc} & & \mathrm{Predicted}\;\mathrm{total}\;\mathrm{Hb}\;\mathrm{in}\;\mathrm{unit}\left(g\right)=\beta 0+\beta 1*\mathrm{Unit}\;\mathrm{volume}\left(\mathrm{mL}\right)\\ & & \;+\;\beta 2*\mathrm{Hb}\;\mathrm{Fingertip}\left(\frac{g}{\mathrm{dL}}\right)+\beta 3*\mathrm{xi}3+\beta 4*\mathrm{xi}4+\beta 5*\\ & & \;\mathrm{xi}5+\beta 6*\mathrm{xi}6 \end{array}$$

*β*0 = (−33.972); *β*1 = 0.263; *β*2 = 0.893;

*β*3 = (−0.386); *β*4 = 0.386; *β*5 = (−0.371); *β*6 = 0.371

*xi*3 = 1 if female, else 0,

*xi*4 = 1 if male, else 0,

*xi*5 = 1 if blood bag system CQ42271 FRESENIUS KABI DEUTSCHLAND GMBH else 0,

*xi*6 = 1 if blood bag system LQT7248LC MACO PHARMA INT. GMBH else 0.

This prediction of total Hb and heme iron content also allows us to estimate Hb and heme iron concentration per milliliter and, thus, to declare these dosing parameters on the pRBC unit label.

### 5.7 Unbiased Assessment of Model Fit in an Independent Data Set Confirms Excellent Performance of MLR Models

**Figure 4:**
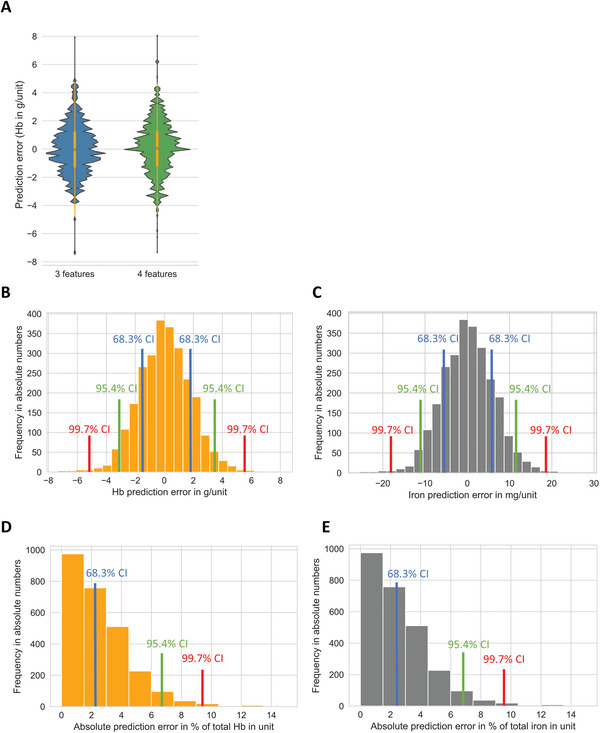
Prediction errors of 3‐ and 4‐feature MLR on the second, independent data set (*n* = 2637). A) Violin plot of prediction errors generated by MLR with 3 (Equation (4)) and 4 features (Equation (5)), respectively. White circle indicates median, orange bar indicates interquartile range (IQR); orange lines stretched from the bar indicate first quartile −1.5 IQR and third quartile +1.5 IQR. B) Histogram of Hb prediction error (MLR with 4 features). C) Histogram of iron prediction error (MLR with 4 features). D) Histogram of relative absolute prediction error to unit Hb in percent for each pRBC unit (MLR with 4 features). E) Histogram of relative absolute prediction error to unit iron in percent for each pRBC unit (MLR with 4 features). Blue lines indicate the limits of the 68.3% confidence interval (mean+/− SD). Green lines indicate the limits of the 95% confidence interval (mean+/− 2*SD). Red lines indicate the limits of the 99.7% confidence interval (mean+/− 3*SD).

For unbiased assessment of our final MLR models with three or four features (Equations (4) and (5)), we used a second, independent data set of 2637 pRBCs randomly collected during June 2020 and May 2021 (Table 1). Both MLR models showed similar performances on this second data set (**Table** **2** and **Figure** **4**A).

**Table 2:** Performance metrics of the models for total Hb content prediction in pRBC units proposed by Arslan et al.,^[^ ^15^ ^]^ Agnihotri et al.^[^ ^10^ ^]^ and Epah et al., assessed on the second, independent data set (*n* = 2637)

	Arslan et al.	Agnihotri et al.	MLR with 4 features	MLR with 3 features
Mean of predicted Hb [g/unit]	74.80	69.56	55.75	55.82
Mean prediction error [g/unit]	−19.0	−13.76	0.05	−0.02
Mean absolute prediction error [g/unit]	19.0	13.77	1.41	1.43
Standard deviation of absolute prediction error [g/unit]	4.22	4.03	1.13	1.13
Mean absolute prediction error [%]	34.71	25.28	2.54	2.58
Standard deviation of absolute prediction error [%]	9.45	8.75	2.04	2.07
Mean squared error	378.71 (recalibrated:^[^ ^43^ ^]^ 17.87)	205.78 (recalibrated:^[^ ^43^ ^]^ 16.53)	3.26	3.32
Standard deviation of mean squared error	159.90	111.47	5.41	5.44
Adjusted coefficient of determination (*R* ^2^)	−8.72 (recalibrated:^[^ ^43^ ^]^ 0.6147)	−4.28 (recalibrated:^[^ ^43^ ^]^ 0.6148)	0.92	0.91

In detail, the MLR model with three features reached an adjusted *R* ^2^ of 0.91, and with four features of 0.92. This is in the range of observed *R* ^2^ values of the nested CV in the first data set with a mean adjusted *R* ^2^ of 0.896 for three features and 0.898 for four features with standard deviations for both of 0.01. The mean prediction error in the second independent cohort for our MLR model with three features was −0.02 g Hb/−0.07 mg iron, and with four features 0.05 g Hb/0.17 mg iron. The limits of the 95% CI were ±3.57 g Hb/12.37 mg iron for MLR with three features and ±3.54 g Hb/12.27 mg iron with four features (Figure 4B,C). The mean absolute prediction error in percentage of the measured Hb/iron content was 2.58% for the MLR with three features, 2.55% for the MLR with four features, and the upper limit of the corresponding 95% CI was 6.55% for both (Figure 4D,E).

### 5.8 MLR Models with both Three and Four Features Achieve Unprecedented Accuracy and Precision for Predicting Hb and Iron Content in pRBC Units

**Figure 5:**
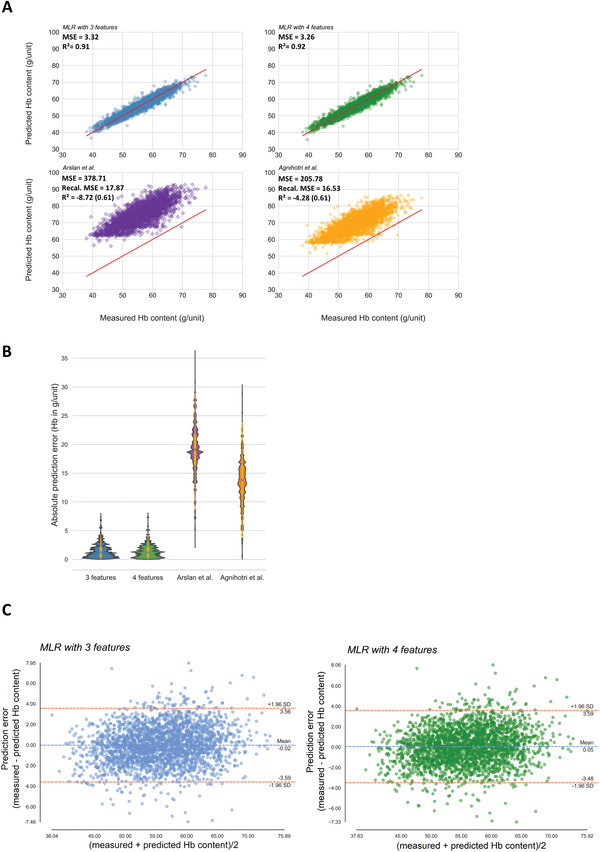
Comparison of the Hb prediction approaches defined in Epah et al., Arslan et al., and Agnihotri et al. on the second, independent data set (*n* = 2637). A) Scatter plot of measured Hb content in g/unit (red) in the second data set versus predicted Hb content using MLR with three features (blue; Equation (4)), four features (green; Equation (5)) and the equations of Arslan et al. (purple) (Equation (2)), and Agnihotri et al. (gold) (Equation (3)). B) Violin plots of absolute prediction errors generated by MLR with three features (blue) (Equation (4)), four features (green) (Equation (5)), Arslan et al. (purple) (Equation (2)), and Agnihotri et al. (gold) (Equation (3)) on the second data set. C) Bland–Altman plot comparing the gold standard (measured Hb) to the MLR with three features (blue; Equation (4)) or four features (green; Equation (5)).

Finally, we compared our both ML‐derived equations with the previous approaches for Hb prediction on our second, independent data set (*n* = 2637). MLR models with three as well as four features predicted Hb content highly accurate with a mean absolute prediction error of 2.58% and 2.55%, respectively, of the Hb content of a given unit (see above), whereas both Arslan et al.^[^ ^15^ ^]^ as well Agnihotri et al.^[^ ^10^ ^]^ overestimated the measured Hb content significantly, with mean absolute prediction errors of 18.997 g Hb/unit (34.71%) and 13.767 g Hb/unit (25.28%) (**Figure** **5**A,B and Table 2).

In addition, the previously published models featured a clearly increased variability of predicted Hb contents compared to our MLR models with three or four features. The low *R* ^2^ values of −8.72 (recalibrated:^[^ ^43^ ^]^ 0.6147) for Arslan et al. and −4.28 (recalibrated:^[^ ^43^ ^]^ 0.6148) for Agnihotri et al. and the corresponding high MSE of 378.71 (recalibrated:^[^ ^43^ ^]^ 17.87) for Arslan et al. and 205.78 (recalibrated:^[^ ^43^ ^]^ 16.53) for Agnihotri et al. obviously illustrate their weak performance, especially in light of *R* ^2^ values above 0.9 and MSE below 3.35 for our models (Table 2). Agreement between measured and predicted Hb content was also graphically assessed via Bland–Altman plots.^[^ ^44^ ^]^ With a mean prediction error of −0.02 g Hb/−0.07 mg iron for MLR with three features and 0.05 g Hb/0.17 mg iron for four features the estimated bias is negligible and 95% of the absolute prediction errors were below 3.59 g Hb/12.37 mg iron (Figure 5C).

### 5.9 Significant Dose and Cost Savings Can Be Achieved by Dosing Chelation Therapy Based on Accurate Predictions of Iron Content in pRBC Units

Demonstrating a use case of a high prediction accuracy for Hb and iron content in pRBC units we modeled the iron load, corresponding Deferasirox doses and costs based on List et al.^[^ ^45^ ^]^ Based on conservative estimations of U.S. drug prices and the minimum, average and maximum iron content per pRBC unit, dose and cost savings of up to 50%, or 616.31$ per day can be achieved when applying the here presented algorithm (**Table** **3**).

**Table 3:** Modelling of iron load and deferasirox doses and costs based on List et al.^[^ ^45^ ^]^

	Mean transfusion rate: 4.1 pRBC units per month over 12 months (Σ = 49 pRBCs per year)	Required amount of Deferasirox 20 mg kg^−1^ bw/d Mean net excretion = 0.329 mg Fe/kg bw/d		Required amount of Deferasirox 40 mg kg^−1^ bw/d Mean net excretion = 0.445 mg Fe/kg bw/d	
Minimum iron content per pRBC unit (133.42 mg)	17.91 mg Fe/day	1088.8 mg/day	387.61 USD/day	1609.9 mg/day	573.12 USD/day
Average iron content per pRBC unit (194.79 mg)	26.15 mg Fe/day	1589.7 mg/day	565.93 USD/day	2350.6 mg/day	836.6 USD/day
Maximum iron content per pRBC unit (276.89 mg)	37.17 mg Fe/day	2259.6 mg/day	804.42 USD/day	3341.1 mg/day	1189.43 USD/day

## 6 Discussion

In this study, we tested more than 6000 pRBC units and found that the pharmaceutically active agent Hb as well as the potential toxic agent heme iron showed a great variability between the units, that is, 38.5 to 79.9 g per unit for Hb and 133.42 to 276.89 mg per unit for iron. Of note, more than a third of these units deviated substantially from the mean Hb content (56.21 g). Such a highly variable Hb and iron content in individual pRBC units contributes to the lack of evidence‐based dosing studies for blood transfusions. Thus, we sought to estimate the Hb content of a given ppRBC unit and therefore tested previously published concepts in a pilot study. However, these approaches, either simply multiplying the volume of the whole blood donation with the measured donor's fingertip Hb value, or additionally subtracting the measured blood volume lost due to leucofiltration, produced highly incorrect estimations with mean prediction errors of 19.55 g Hb/unit (30.8%) and 14.35 g Hb/unit (26.4%), respectively. We asked whether applying a more sophisticated modelling approach and taking additional parameters into account could provide more accurate and precise estimates without compromising the integrity of the pRBCs. We therefore considered all thirteen parameters being routinely collected during blood donation, pRBC production, and quality testing. Using a training data set of 6058 pRBCs we tested eight different ML models and subsequently assessed the selected models in a second independent cohort of 2637 pRBCs. With our novel ML approach, we predicted the individual hemoglobin and iron content of a given pRBC unit with a mean absolute prediction error of ≤1.43 g hemoglobin/4.96 mg iron and a *R* ^2^ > 0.9 in the second cohort, thereby highly outperforming the previous attempts. We further showed that applying this algorithm could lead to substantial dose and cost reduction of iron chelation therapy and enables for the first time true Hb dosing studies for blood transfusions in the clinic.

In 2005 Robertson Davenport wondered in disbelief “Who would order a drug today without knowing the actual dose?”, but at the same time he had to admit that this was,^[^ ^46^ ^]^ and still is, the current clinical standard for blood transfusions. As mentioned above the current clinical practice for adults is to dose pRBCs in units. However, the transfusion effect, that is, Hb increase in patient's blood, varies significantly depending on several factors such as patient weight that correlates inversely to the Hb increase.^[^ ^47^ ^]^ Moreover, we show here that the active agent of pRBCs, that is, Hb, varies substantially between pRBC units. This is even true for units of identical volume (Figure S2, Supporting Information). Thus, specifying total Hb content and concentration of individual pRBC units are key factors to define the actual dose of the drug. Precise Hb‐based dosing would allow for the first time to conduct dose‐effect studies for pRBC applications in different patient cohorts such as in surgery and hematology.

As per international consensus, implementation of PBM programs for various clinical scenarios warrants further high‐quality research.^[^ ^48^ ^]^ Clinical recommendations for RBC transfusion thresholds and PBM program implementations to improve RBC utilization are mainly based on low to moderate certainty in the evidence of actual transfusion effects.^[^ ^48^ ^]^

Recent studies investigated the impact of blood donor, pRBC product, and patient traits on RBC transfusions' clinical efficacy. They found that donor genetics, patient characteristics such as sex, body mass index, ethnicity and age, as well as some product features, for example, collection method or gamma irradiation, affected the transfusion effectiveness.^[^ ^49^, ^50^ ^]^ However, these highly valuable studies, having retrospectively analyzed the transfusion effects of more than 140 000 pRBCs, could not consider their individual Hb content.

Thus, it is reasonable to hypothesize that the unknown individual Hb concentration in pRBC units substantially contributes to the current uncertainty. Our herein presented concept now allows the design of PBM studies considering accurate Hb dosing instead of neglecting the Hb concentrations of the applied pRBC units. Such precision transfusion medicine would not only supply the individual patient with the actually required Hb dose, but would also enable blood banks to manage and monitor their pRBC inventory more precisely. The herein proposed shift from units to actual Hb doses could also increase the general availability of pRBCs including O negative products. This is of particular importance as, due to demographic changes, the future demand of RBC products could supersede supply if blood centers would not adjust their operations.^[^ ^51^ ^]^

Moreover, Hb‐adjusted dosing could prevent unnecessary transfusions, hereby reducing the risk of infections, immunizations, wrong‐patient blood transfusion errors and transfusion‐associated circulatory overload,^[^ ^52^ ^]^ where specifically the latter comes in risk groups at a frequency of up to 1:12.^[^ ^3^ ^]^

Previously, other groups have thought about strategies to estimate total Hb content in pRBC units, but used oversimplified approaches and did not compare their predictions to true Hb contents at all,^[^ ^15^, ^18^ ^]^ or only to the overall Hb content distribution of the analyzed pRBC cohort.^[^ ^10^ ^]^ Despite its limitations, the first of these aforementioned studies showed that a transfusion regime considering Hb content estimations could decrease the numbers of transfused pRBC units for some patients.^[^ ^15^, ^18^ ^]^ The low accuracy of the second study is specifically highlighted by the substantial (20%) difference between the standard deviation of the calculated Hb content per unit and the standard deviation of the actual Hb content.^[^ ^10^ ^]^ To the best of our knowledge we herein present for the first time highly reliable, non‐invasive Hb content/concentration predictions of individual pRBC units. Notably, our Hb prediction ML concept was validated by comparing the individual predicted to the measured Hb content of the respective pRBC unit. Thus, we could show that our approach clearly outperformed the previous concepts as highlighted by a higher prediction accuracy and precision on the second, independent data set (Figure 4A,B and Table 2). This is of clinical importance, as relying on equations that strongly overestimate actual Hb content of a given pRBC unit could lead to undertransfused patients with potential severe consequences. We show that relatively few variables are sufficient for a highly accurate estimation suggesting a viable solution to a complex problem.

Several other ML models performed virtually as well as the MLR in our analysis (Figure 3A, Table S1, Supporting Information), but we selected the MLR for its easier applicability and interpretability,^[^ ^50^ ^]^ thereby considerably facilitating the implementation for blood banks and its transmission into the clinic. Notably, MLR could predict pRBC units of an entire subsequent year with an acceptable margin of error (Figure S5, Supporting Information), and the possible clinical benefit of our proposed MLR based prediction method is reinforced by the high model performance with negligible mean prediction error and an acceptable margin of error on our second, independent data set (Figure 5 and Table 2).

Besides the comprehensible additional features Hb fingertip and sex for total Hb content prediction (Figures S2 and S3, Supporting Information), we identified another feature, that is, blood bag system type, which slightly, but not significantly, increased the prediction performance in the first data set and in the second, independent data set (Figures 3C and 4A, and Table 2). The mechanisms of how the variable blood bag system type affects the small differences in Hb/iron prediction remain unclear. Although the blood bag systems do not differ substantially in composition of anticoagulant and RBC nutrient solution, they vary in their valve systems and overall bag diameter, as well as in their tube length and tube diameter. These slight variations may impose different physical forces on the blood in the collection system during production process that might affect the amount of pRBCs in the final bag. Performance did not substantially differ between the production sites or seasons, such supporting the robustness of the prediction algorithm (Figure S4, S5, Table S2, Supporting Information). In our data set we could also not detect an impact of the donor's ABO blood group or Rhesus D phenotype on the prediction precision of the algorithm (Figures S6 and S7, Supporting Information). This, however, may need further verification with other data sets due to the limited amount of quality control data of pRBCs from donors with non‐B blood groups and Rh D negative phenotype in our data set.

Recent studies showed that post‐transfusion Hb increments decrease and extravascular hemolysis increases with pRBC unit storage age.^[^ ^49^, ^50^, ^53^ ^]^ This could indicate a decreasing drug (i.e., pRBC) effectiveness over time, even with appropriate storage handling. In our study we investigated Hb content and its predictability in pRBC units at the beginning of their storage life. Therefore, future studies may consider expanding our approach by additionally incorporating the storage age and the irradiation status of the pRBC product into the algorithm.

We herein report both equations to enable other production sites to apply locally available features. Relevant variations may occur in different regions worldwide, and specific co‐influencing factors in other donor cohorts might warrant further adaptation, for example, based on local quality control data, to keep the accuracy of the predictions. As such our approach, validated with pRBCs from multiple production sites including multiethnic metropolitan agglomerations as well as rural areas, might be applicable to blood banks worldwide with a sufficient amount of local adjustment including donor cohort‐specific data. Because the globally mandatory individual fingertip Hb test prior to collection is relevant for the prediction model and the measurement of Hb concentration is not influenced by ethnicity, we do not expect the donors’ ethnicity to have a significant impact on the prediction accuracy, but we cannot rule it out at this time. Moreover, different methods of the donor's Hb assessment, for example, pricking the ear lobe rather than the fingertip, could affect the accuracy of the Hb measure in the donor's blood and thus the performance of our final models.^[^ ^54^ ^]^ Therefore, further studies are needed to determine if our approach can be directly transferred to blood banks globally, or whether, and to which extent, local adaptations are required.

To facilitate the use of the MLR model with three features we developed a web application (Figure S8, Supporting Information) to predict single pRBC units with a slider (Figure S8A, Supporting Information) as well as entire data sets through an upload of a comma‐separated values file (Figure S8F, Supporting Information). In addition, user‐specific data sets can be uploaded and used for retraining of the MLR (Figure S8B, Supporting Information). This web application can be accessed through streamlit (https://epahjeremy‐prbc‐prediction‐hbprediction‐dceyew.streamlitapp.com) and the corresponding source code through github (https://github.com/epahjeremy/prbc‐prediction).

Applying the herein presented concept could enable blood banks to support clinicians in replacing their current transfusion regimens by accurate Hb dosing that not only decrease the above mentioned transfusion risks, but would also lead to improved monitoring of transfusion associated iron uptake, which is highly variable from 6 to 41 mg per day.^[^ ^55^ ^]^ We found that more than every third (38%) of the here tested pRBC units substantially deviated (≥±15%) from the mean Hb and iron content (56.21 g/194.79 mg) (Figure 1A). Given the annual production for Germany in 2017 (4.0 million pRBC units^[^ ^56^ ^]^) these 38% represent the considerable amount of 1.52 million pRBC units.

In patients with thalassemia who do not receive transfusions, iron absorption from the intestine, triggered by expansion of red cell precursors in the bone marrow, is increased, but transfusing RBCs to an Hb above 9 g dL^−1^ can avoid this expansion.^[^ ^9^, ^55^ ^]^ However, in transfusion‐dependent thalassemia, the contribution of dietary iron to the total iron load is minimal compared to the effect of pRBC transfusions which increase iron stores to many times the norm unless chelation treatment is provided.^[^ ^9^ ^]^ During pRBC production the plasma is removed after centrifugation leaving only packed RBCs and iron‐free preservation solution in the product for transfusion. As shown previously, heme iron is the only relevant iron source in pRBCs.^[^ ^4^, ^57^ ^]^ The effects of iron chelation drugs have been studied for decades, and List et al. showed that chelation drug therapy can decrease the most toxic NTBI fraction, that is, labile plasma iron.^[^ ^45^ ^]^ Referring to this hallmark study we calculated, based on the minimal, average and maximal iron content of the pRBCs that we investigated in our study, the respective daily iron load and chelation drug doses and costs (Table 3). Even though RBCs can also contain negligible amounts of chelated zinc protoporphyrin^[^ ^58^ ^]^ it is well established that 1 mol Hb contains 4 mol iron.^[^ ^59^ ^]^ Iron measurements performed in the laboratory including both of total or labile iron, often require extensive sample handling and specialized instruments, that are time consuming and laborious. Moreover, there is minimal to no overlap between total iron and labile iron quantification methodologies, that is, requiring entirely separate protocols, techniques and instruments.^[^ ^60^ ^]^ In addition, and most importantly, relying on laboratory iron measure would add, in addition to fingertip Hb and pRBC volume measures, a third analytic methodology that comes with its own variability, thus, compromising the accuracy of the prediction. Thus, applying the stoichiometric calculation based on the pRBC's Hb content is the most exact determination of the respective pRBC's iron content. Hereby, we show that it is possible to precisely adjust a chelation drug dose to daily transfusion‐related iron load and to better control the economic impact of this costly therapy.

Since smartphones are widely adopted,^[^ ^61^ ^]^ we here propose a simple concept for a smartphone application to facilitate iron monitoring for both physicians and patients (Figure S9, Supporting Information). Forgetfulness and a lack of social support could affect compliance to oral medication in chronically ill pediatric patients.^[^ ^62^ ^]^ One possible future application of our method in pediatric patients could be in sickle cell disease (SCD), where many European countries, the U.S.A. and Canada implemented newborn SCD screening programs.^[^ ^63^, ^64^ ^]^ Our method would not only allow to non‐invasively monitor iron dosage and chelation therapy (Figure S9F, Supporting Information), but could also increase therapy adherence through gamification^[^ ^65^, ^66^ ^]^ (Figure S9G, Supporting Information) and fewer invasive procedures.^[^ ^67^ ^]^

Blood donors could also benefit from the here presented approach. Protecting those is paramount for the entire health care system for ethical reasons and to ensure the supply of blood products.^[^ ^68^ ^]^ It is well documented that anemia from iron deficiency negatively effects learning and educational achievement.^[^ ^69^, ^70^, ^71^ ^]^ Notably, increased risk for iron deficiency can last up to a year after donation^[^ ^72^ ^]^ and frequent blood donations can lead to iron deficiency even in adults.^[^ ^16^ ^]^ A recent study reported accelerated iron stores and Hb depletion in teenage blood donors^[^ ^73^ ^]^ highlighting the need for improved measures to protect vulnerable young donors from donation‐induced iron deficiency and iron deficiency anemia.^[^ ^73^ ^]^ Thus, advanced documenting and monitoring could increase donor safety and limit donation side effects.

Our concept allows not only for applying a specific Hb dose with minimal iron burden, hereby also optimizing the chelation medication, but could also improve blood donor safety. This highlights the relevance of our findings for both patient and donor blood management and the clinical transfusion practice toward a personalized therapy approach.

## Conflict of Interest

The authors declare no conflict of interest.

## Author Contributions

J.E. and R.S. designed the study, analyzed the data, and wrote the manuscript. I.G., J.D., E.H., and D.L. collected data and reviewed the manuscript. S.W., C.G., M.S., and E.S discussed the results and reviewed the manuscript. All authors had full access to all the data of the study and accept responsibility to submit for publication.

## Supporting information

Supporting Information

## Data Availability

The data that support the findings of this study are available from the corresponding author upon reasonable request.
