# Supplementary material for: From Unit to Dose: A Machine Learning Approach for Precise Prediction of Hemoglobin and Iron Content in Individual Packed Red Blood Cell Units
Source: Adv Sci (Weinh). 2022 Nov 4;9(36):2204077. doi: 10.1002/advs.202204077 (PMC9798979; doi:10.1002/advs.202204077)
Supplement: Supplementary file 1 — Supporting Information [file ADVS-9-2204077-s001.pdf]

Supporting Information for

From unit to dose:
A machine learning approach for precise prediction of hemoglobin and iron content in individual packed red blood cell units

Jeremy Epah[1], Ilay Gülec[1], Stefan Winter[2], Johanna Dörr[1], Christof Geisen[1], Eva Haecker[1], Dietmar Link[1], Matthias Schwab[2,3,4], Erhard Seifried[1] and Richard Schäfer[1,5,6]

[1]Institute for Transfusion Medicine and Immunohaematology
German Red Cross Blood Donor Service Baden-Württemberg-Hessen gGmbH
Goethe University Hospital, Frankfurt am Main, Germany

[2]Dr. Margarete Fischer-Bosch Institute of Clinical Pharmacology, Stuttgart, Germany and University of Tübingen, Tübingen, Germany

[3]Departments of Clinical Pharmacology, Pharmacy and Biochemistry
University of Tübingen, Tübingen, Germany

[4]Cluster of Excellence iFIT (EXC 2180) „Image-Guided and Functionally Instructed Tumor Therapies", University of Tübingen, Germany

[5]Institute for Transfusion Medicine and Gene Therapy
Medical Center - University of Freiburg, Germany

[6]Center for Chronic Immunodeficiency (CCI)
Medical Center - University of Freiburg, Germany

Correspondence

PD Dr. Dr. Richard Schäfer, M.D.
Institute for Transfusion Medicine and Gene Therapy
Medical Center - University of Freiburg
Hugstetter Str. 55, 79106 Freiburg, Germany
richard.schaefer.md@gmail.com

Dr. Jeremy Epah, M.D.
Institute for Transfusion Medicine and Immunohematology
Johann Wolfgang Goethe-University Hospital
Sandhofstrasse 1, 60528 Frankfurt, Germany
jeremy@epah.de

Extracting the following equations from their respective publications

To simplify the calculation, we wrote down all equations using liters as unit. This means that data displayed in milliliters or deciliters must first be converted to liters (divide by 1000, multiply by 10).Agnihotri *et al.* do not converted their units but simply divide by 100 (see below). This gives the correct numerical result but does lead to some confusion in regards to the respective units. We therefor converted everything in to liters first.

Calculating the Hb content in pRBC units as proposed by Arslan *et al.*[1] and Atilla *et al.* [2]

In their paper, in which *WB* stands for whole blood, Arslan *et al.* state that (Arslan O *et al.*, Transfusion 44, 485–488(2004), section "Material and Methods") *„Initial assessment of donor Hb concentration was obtained by finger-stick puncture as part of the routine procedure at the donor sessions. Calibrated HemoCue hemoglobinometer was used for Hb determination. [...] Volume of WB collected during phlebotomy and the pre-transfusion Hb value of the donors were all recorded on the software as part of a routine procedure. [...] Hemosoft calculated the total Hb content of the units by multiplying the volume with the Hb value of the donors."*

From this we, concluded that Arslan *et al.* proposed to compute the total Hb content per unit as the product of the whole blood donation volume and the (calibrated) Hb fingertip value of the donors. Consequently, we presented the following equation in our manuscript for calculation of the Hb content in pRBC units according to Arslan *et al.*:

$$\text{Predicted total Hb in unit (g)} = \text{Whole blood donation } (L) * \text{ Fingertip Hb } \left(\frac{\text{g}}{L}\right) \qquad (2)$$

Calculating the Hb content in pRBC units as proposed by Agnihotri *et al.* [3]

Using THb as abbreviation for the total Hb content (THb), Agnihotri *et al.* state (Agnihotri N *et al.*Blood transfusion 12, 520–526 (2014) in the paragraph "Total haemoglobin content in the red blood cell units":

"The mathematical calculation of the THb was done as follows:

First, total Hb collected in these RBC units was calculated. The pre-donation Hb of the donor (of these units was used for this purpose as follows:

Total Hb collected, in grams ***(A)*** *= [Donor Hb (in g/dL) × blood volume collected (in mL)/100]"*

Note that here, Donor Hb means the pre-donation Hb of the donor, i.e. the Fingertip Hb. The above mentioned paragraph carries on with:

"The blood loss during leucofiltration and other procedures (e.g. transfer between satellite bags, tubing, etc.) was calculated prospectively on 50 RBC units by subtracting post-leucofiltration RBC volume from pre-leucofiltration RBC volume. The Hb lost was calculated as follows:

Hb lost during processing, in grams
(B) *= [Donor Hb(in g/dL) × blood volume lost due to processing(in mL)/100]*

THb in these RBC units was then mathematically calculated as follows: Mathematically calculated THb per unit (in grams) ***A - B****"*

In the paragraph "Total haemoglobin content in the red blood cell units" of their paper they further state: *"Prospective testing of 50 RBC units found that, at our centre, an average of 35±2.3 mL blood was lost during the preparing of a unit of leucofiltered RBC from the whole blood."*

We therefore computed the predicted total Hb content per unit according to Agnihotri et al. as **A** – **B**:

Donor Hb (in g/dL) * blood volume collected (in mL)/100 – Donor Hb in g/dL * 0.35mL, or with the slightly different notation in our manuscript:

$$Predicted\ total\ Hb\ \ in\ unit\ (g) = Whole\ blood\ donation\ (L) * Fingertip\ Hb\ \left(\frac{g}{L}\right) - 0.035\ (L) * Fingertip\ Hb\ \left(\frac{g}{L}\right) \quad (4)$$

Example:

500 mL = 0.5 L whole blood donation

$12\ \frac{g}{dL} = 120\ \frac{g}{L}$ Fingertip Hb

Insert into equation 8 results in: $0.5\ L * 120\ \frac{g}{L} - 0.035\ L * 120\ \frac{g}{L} = 55.8\ g$

Calculating the dose of Deferasirox and price required to excrete yearly iron load due to pRBC unit transfusions (based on List *et al.* [4])

$$Annually\ transfused\ pRBC\ units\ =\ 4.1\ pRBC\ units/month\ *\ 12\ months \tag{S1}$$

$$Iron\ dosage/day\ =\ \frac{iron\ content\ of\ pRBC\ units\ (mg) * annually\ transfused\ pRBC\ units}{365\ days} \tag{S2}$$

$$Required\ Deferasirox\ dose/day\ =\ \frac{iron\ dosage/day}{mean\ net\ excretion\,\frac{Fe}{kg}\,bw/d} * Deferasirox\ dosage\,\frac{mg}{kg}\,bw/d \tag{S3}$$

$$Costs\ of\ Deferasirox/day = Required\ Deferasirox\ dose/day * \frac{0.356\ \$}{mg\ Deferasirox} \tag{S4}$$

In this study the mean RBC transfusion rate was 4.1 RBC units per month over 12 months.

Mean net excretion rates based on the respective dose were obtained from P.L. Carver, Ed., Essential Metals in Medicine: Therapeutic Use and Toxicity of Metal Ions in the Clinic, De Gruyter 2019:

“deferasirox (10, 20, and 40 mg/kg/day) was able to induce net iron excretion. (0.119, 0.329, and 0.445 mg Fe/kg body weight/d, respectively)” [5]

Calculating the price of 1 mg Exjade (Deferasirox) in the United States:

500 mg Exjade oral tablet, dispersible
from **$5,345.26** for 30 tablets

Quantity	Per unit	Price
30	$178.18	$5,345.26

https://www.drugs.com/price-guide/exjade (23.04.2022)

$$0{,}35636\$/mg\ Deferasirox = \frac{178.18\$}{500\ mg}$$

Supporting Tables

Table S1

Model	***R^2***			***MSE***		
	Mean	***Median***	***SD***	***Mean***	***Median***	***SD***
MLR	***0.903***	***0.903***	***0.009***	***3.903***	***3.895***	***0.312***
SVM	***0.902***	***0.902***	***0.009***	***3.958***	***3.955***	***0.322***
LGBMR	***0.900***	***0.900***	***0.009***	***4.006***	***4.013***	***0.299***
RANSAC	***0.898***	***0.898***	***0.010***	***4.120***	***4.099***	***0.412***
DecTree	***0.885***	***0.885***	***0.010***	***4.643***	***4.646***	***0.340***
RF	***0.881***	***0.881***	***0.009***	***4.798***	***4.776***	***0.379***
NN	***0.877***	***0.887***	***0.036***	***4.880***	***4.439***	***1.388***
KNN	***0.875***	***0.876***	***0.010***	***5.011***	***5.004***	***0.380***

Mean, median and standard deviation (SD) of the coefficient of determination (R^2) and mean squared error (MSE) for eight different ML models for Hb prediction on the first data set (n=6,058), using all 13 features as input variables; 50 times repeated nested ten-fold CV.

Table S2

3 features	prediction error				absolute prediction error						
**PRODUCTION SITE**	**median**	**mean**	**sd**		**median**	**mean**	**sd**		**mse**	**R^2**	**Adj R^2**
**A**	0.10	0.19	1.87		1.27	1.47	1.17		3.53	0.90	0.90
**B**	-0.03	0.19	1.98		1.29	1.53	1.30		4.02	0.87	0.87
**C**	0.23	0.19	1.77		1.19	1.42	1.09		3.19	0.91	0.91
**D**	-0.17	-0.12	1.78		1.17	1.40	1.12		3.20	0.91	0.91
**E**	-0.31	-0.29	1.83		1.19	1.44	1.16		3.43	0.90	0.90

4 features	prediction error				absolute prediction error						
**PRODUCTION SITE**	**median**	**mean**	**sd**		**median**	**mean**	**sd**		**mse**	**R^2**	**Adj R^2**
**A**	0.20	0.18	1.86		1.16	1.45	1.17		3.48	0.90	0.90
**B**	0.16	0.33	1.98		1.28	1.53	1.30		3.95	0.87	0.87
**C**	0.21	0.18	1.75		1.19	1.40	1.06		3.08	0.92	0.91
**D**	-0.05	-0.02	1.779		1.15	1.38	1.12		3.16	0.91	0.91
**E**	-0.13	-0.14	1.8266		1.15	1.42	1.15		3.35	0.90	0.90

Prediction error, absolute prediction error, mean squared error (mse), coefficient of determination (R^2) and adjusted R^2 (Adj R^2) for each production site of MLR for Hb prediction on the second data set (n=2,637), using three or four features as input variables; sd=standard deviation.

Legends for the Figures in the Supporting Information

Figure S1

A

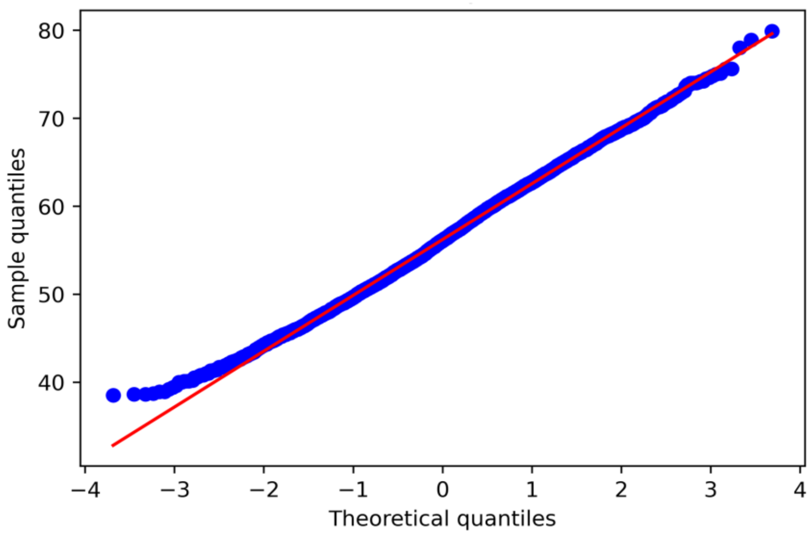

B

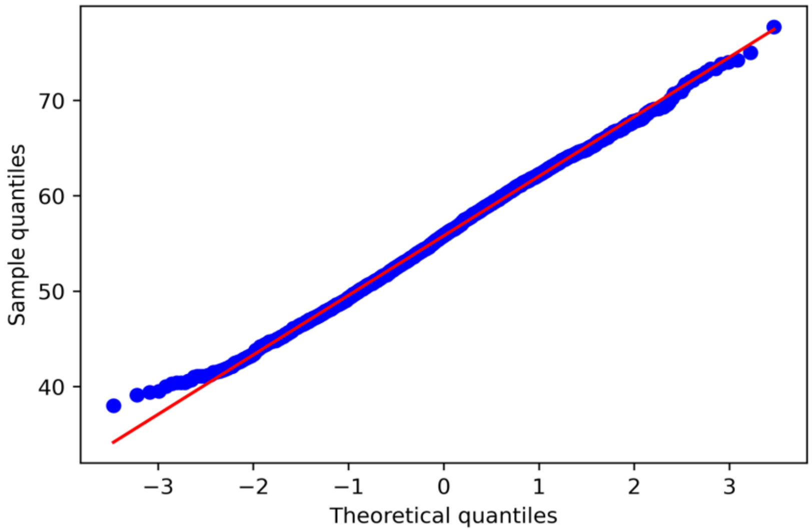

Figure S1 Normal Q-Q plots of total Hb content in g/unit

Normal Q–Q plots of total Hb content in g/unit in the first data set (n=6,058) (A) and second data set (n=2,637) (B). The measured Hb data fits well to a normal distribution (red line).

Figure S2

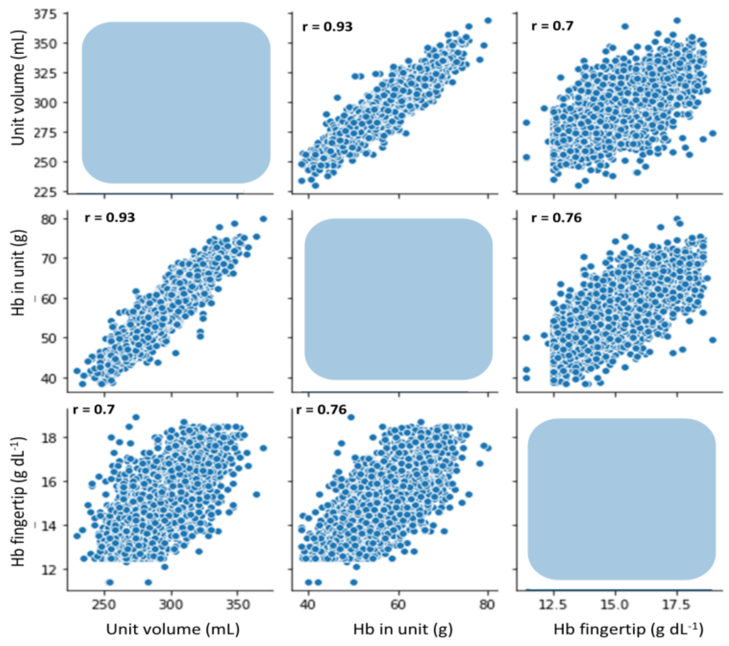

Figure S2 Correlation analysis of Unit volume (mL), Hb in unit (g), and Hb fingertip (g dL^{-1}) on the first data set (n = 6,058)

Scatter plot matrix displaying pairwise relationships and Pearson correlation coefficients (r) of Unit volume (mL), Hb in unit (g), and Hb fingertip (g dL^{-1}) in the first data set.

Figure S3

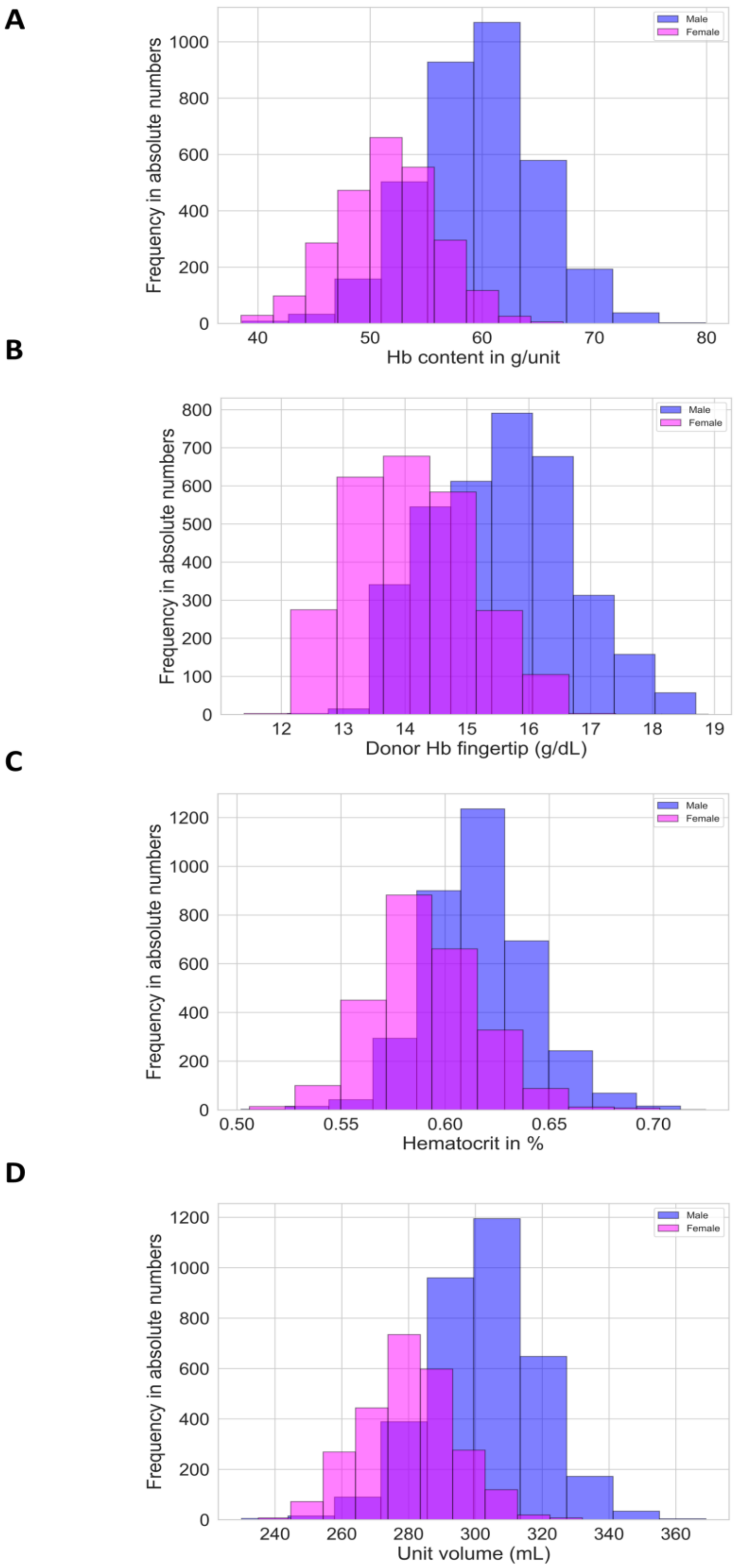

Figure S3 Impact of donor´s sex on unit Hb, Hb fingertip, Hct and unit volume in the first data set (n = 6,058)

Sex differences regarding

A: Target value (total Hb content in g/unit).

B: Hb fingertip (g dL^{-1})

C: Hematocrit per Unit (%)

D: Unit Volume (mL)

Figure S4

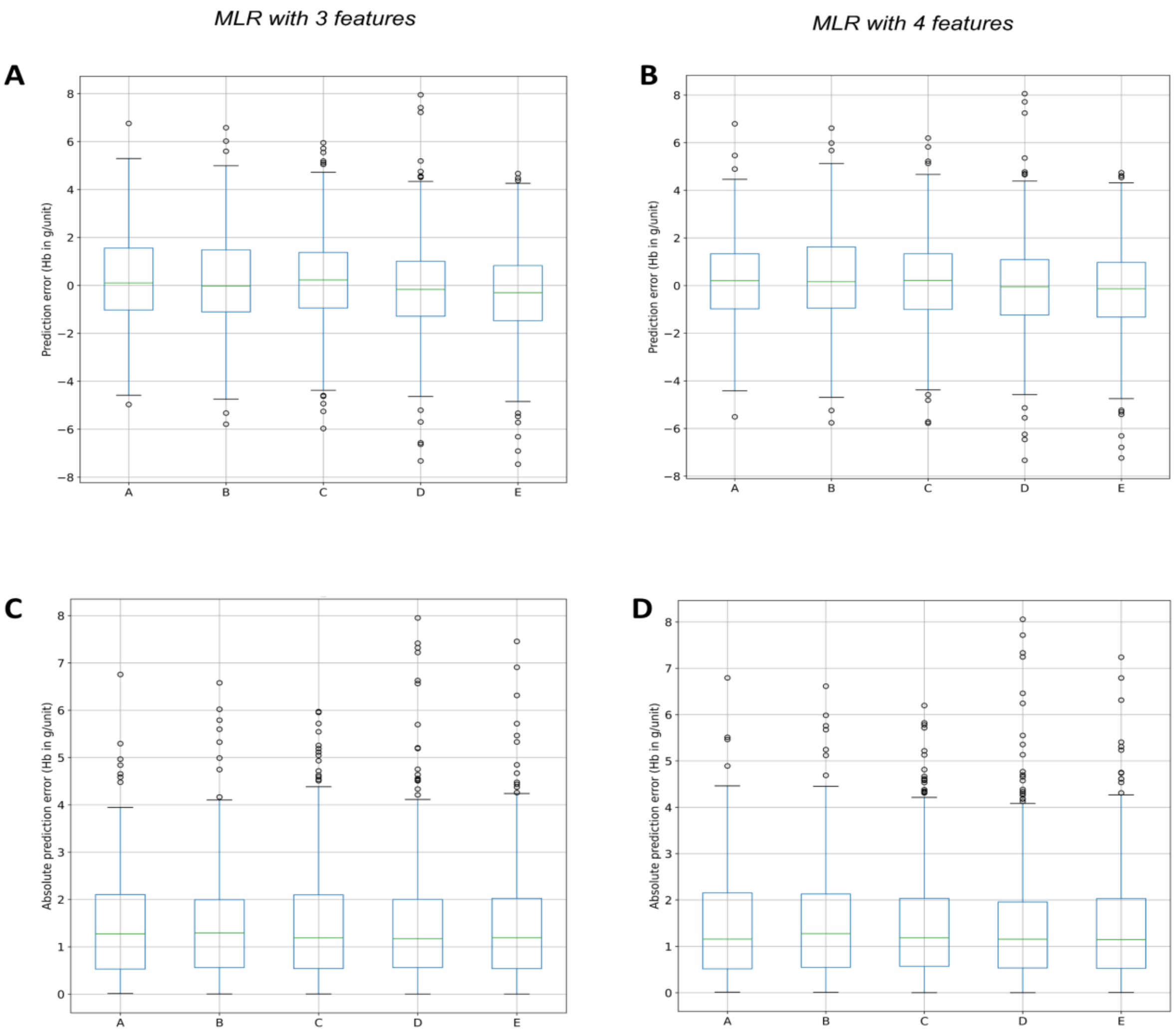

Figure S4 Impact of production site on MLR Hb prediction in the second data set (n=2,637)

Boxplots of Hb prediction grouped by production site regarding

A: Prediction error (Hb in g/unit) in MLR with 3 features

B: Prediction error (Hb in g/unit) in MLR with 4 features

C: Absolute prediction error (Hb in g/unit) in MLR with 3 features

D: Absolute prediction error (Hb in g/unit) in MLR with 4 features

n = 2,637.

Green lines indicate medians; upper and lower box borders indicate interquartile range (25. – 75. percentile); error bars: 99.7% CI; circles: outliers.

Figure S5

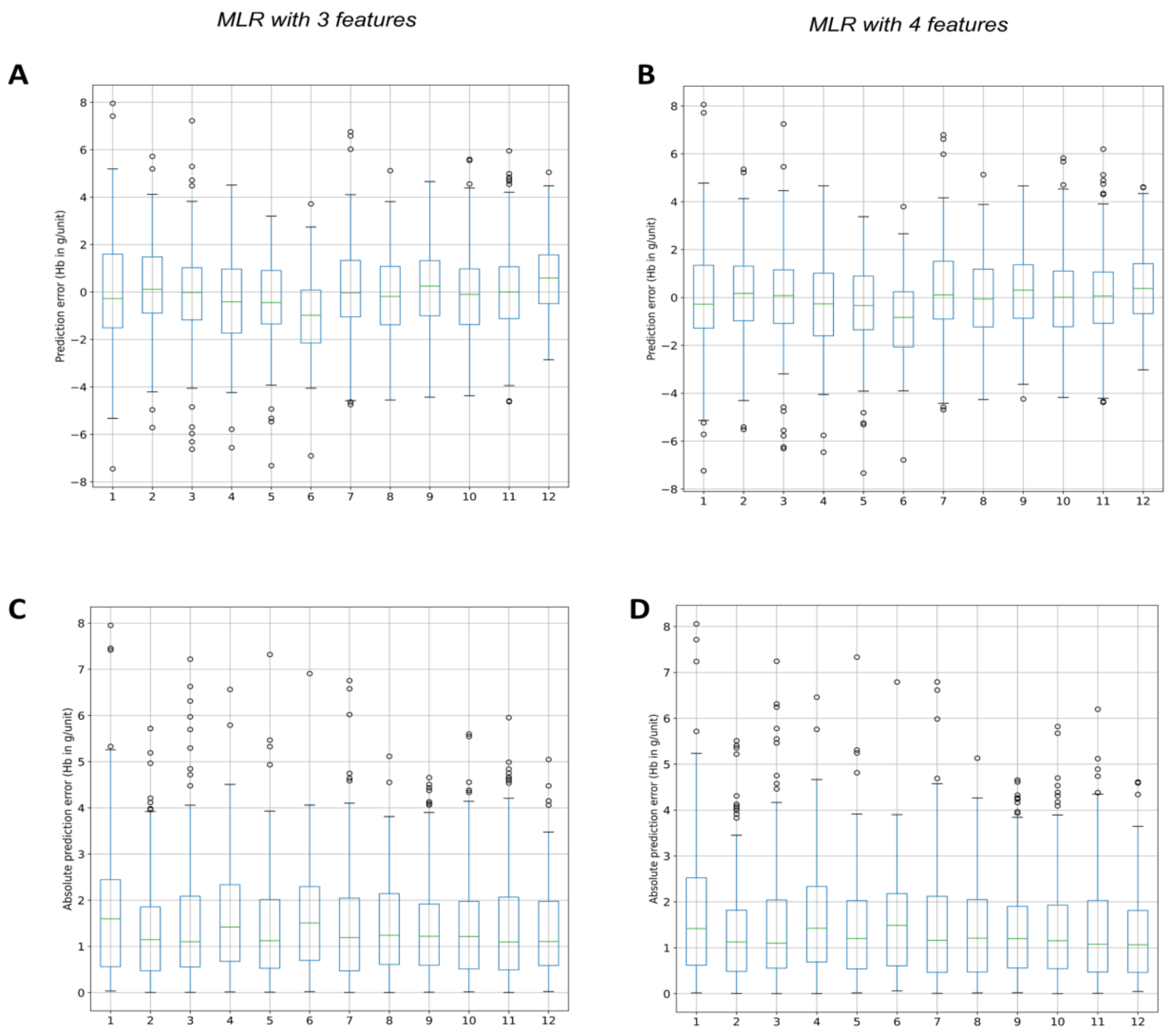

Figure S5 Impact of production month on MLR Hb prediction in the second data set (n=2,637)

Boxplots of Hb prediction grouped by production month regarding

A: Prediction error (Hb in g/unit) in MLR with 3 features

B: Prediction error (Hb in g/unit) in MLR with 4 features

C: Absolute prediction error (Hb in g/unit) in MLR with 3 features

D: Absolute prediction error (Hb in g/unit) in MLR with 4 features

n = 2,637.

Green lines indicate medians; upper and lower box borders indicate interquartile range (25. – 75. percentile); error bars: 99.7% CI; circles: outliers.

The numbers 1 to 12 represent the months from January to December in their calendrical order.

Figure S6

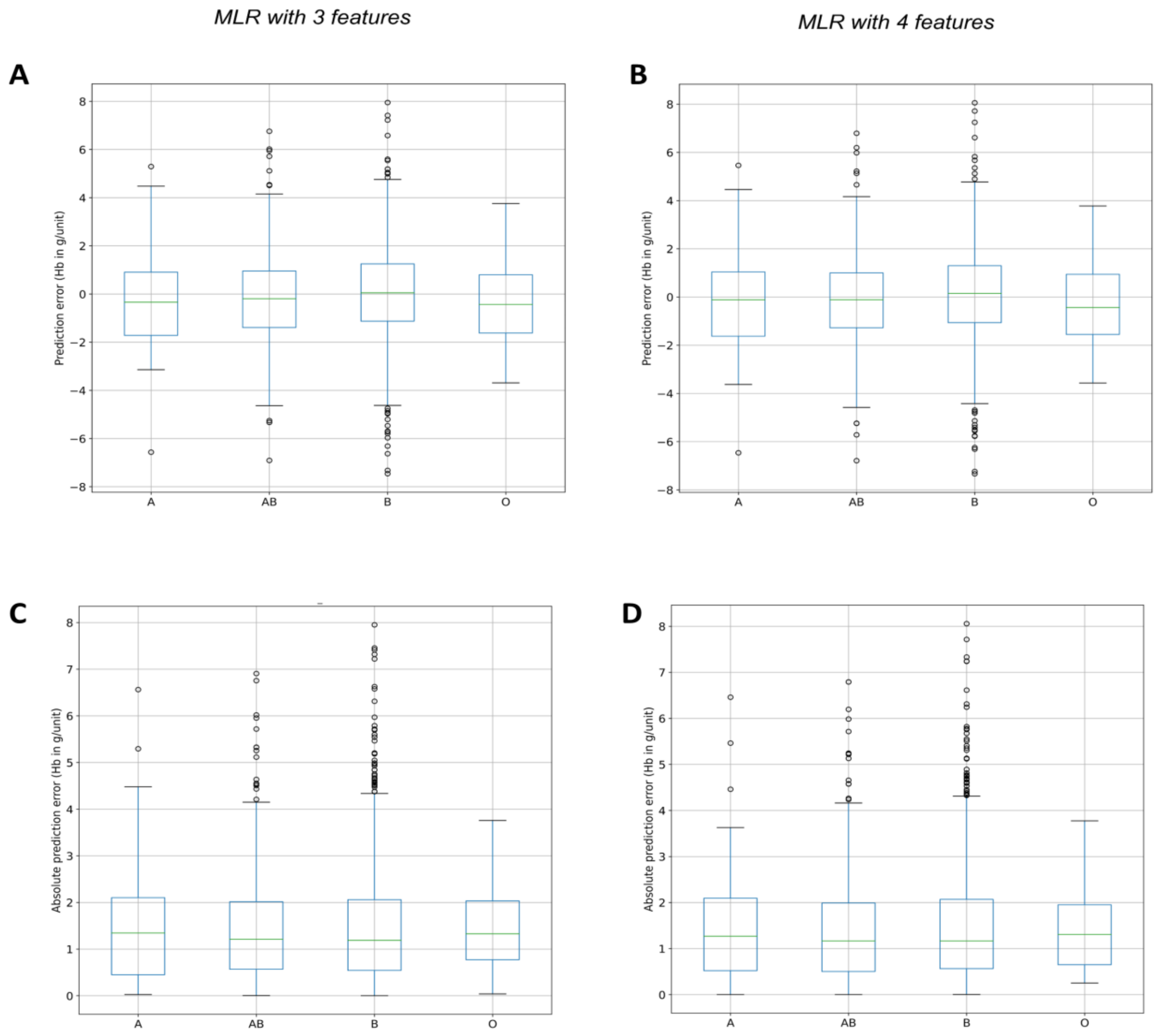

Figure S6 Impact of ABO blood group on MLR Hb prediction in the second data set (n=2,637)

Boxplots of Hb prediction grouped by ABO blood group regarding

A: Prediction error (Hb in g/unit) in MLR with 3 features

B: Prediction error (Hb in g/unit) in MLR with 4 features

C: Absolute prediction error (Hb in g/unit) in MLR with 3 features

D: Absolute prediction error (Hb in g/unit) in MLR with 4 features

n = 2,637.

Green lines indicate medians; upper and lower box borders indicate interquartile range (25. – 75. percentile); error bars: 99.7% CI; circles: outliers.

Figure S7

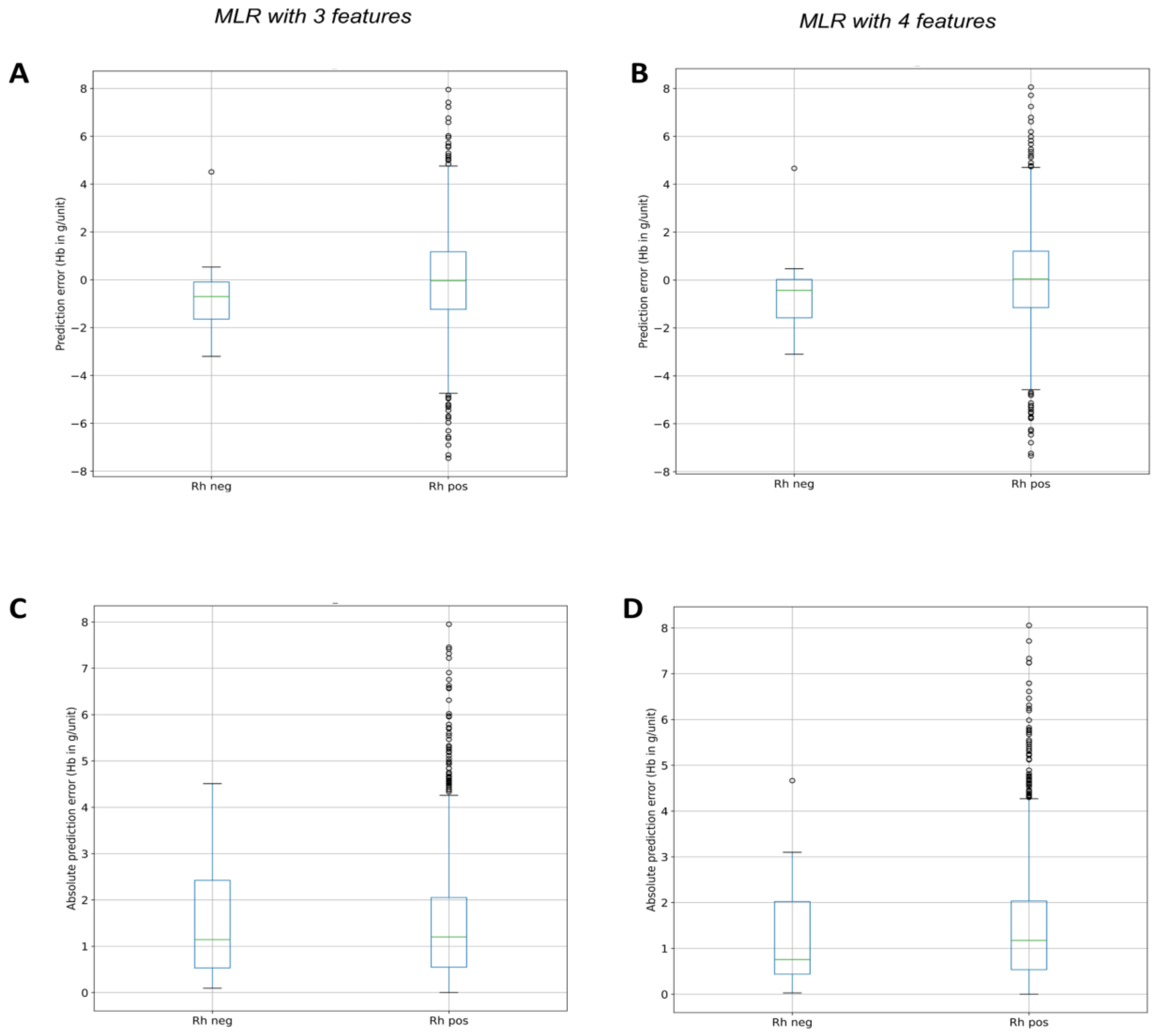

Figure S7 Impact of Rh D phenotype on MLR Hb prediction in the second data set (n=2,637)

Boxplots of Hb prediction grouped by Rh D phenotype regarding

A: Prediction error (Hb in g/unit) in MLR with 3 features

B: Prediction error (Hb in g/unit) in MLR with 4 features

C: Absolute prediction error (Hb in g/unit) in MLR with 3 features

D: Absolute prediction error (Hb in g/unit) in MLR with 4 features

n = 2,637.

Green lines indicate medians; upper and lower box borders indicate interquartile range (25. – 75. percentile); error bars: 99.7% CI; circles: outliers.

Figure S8

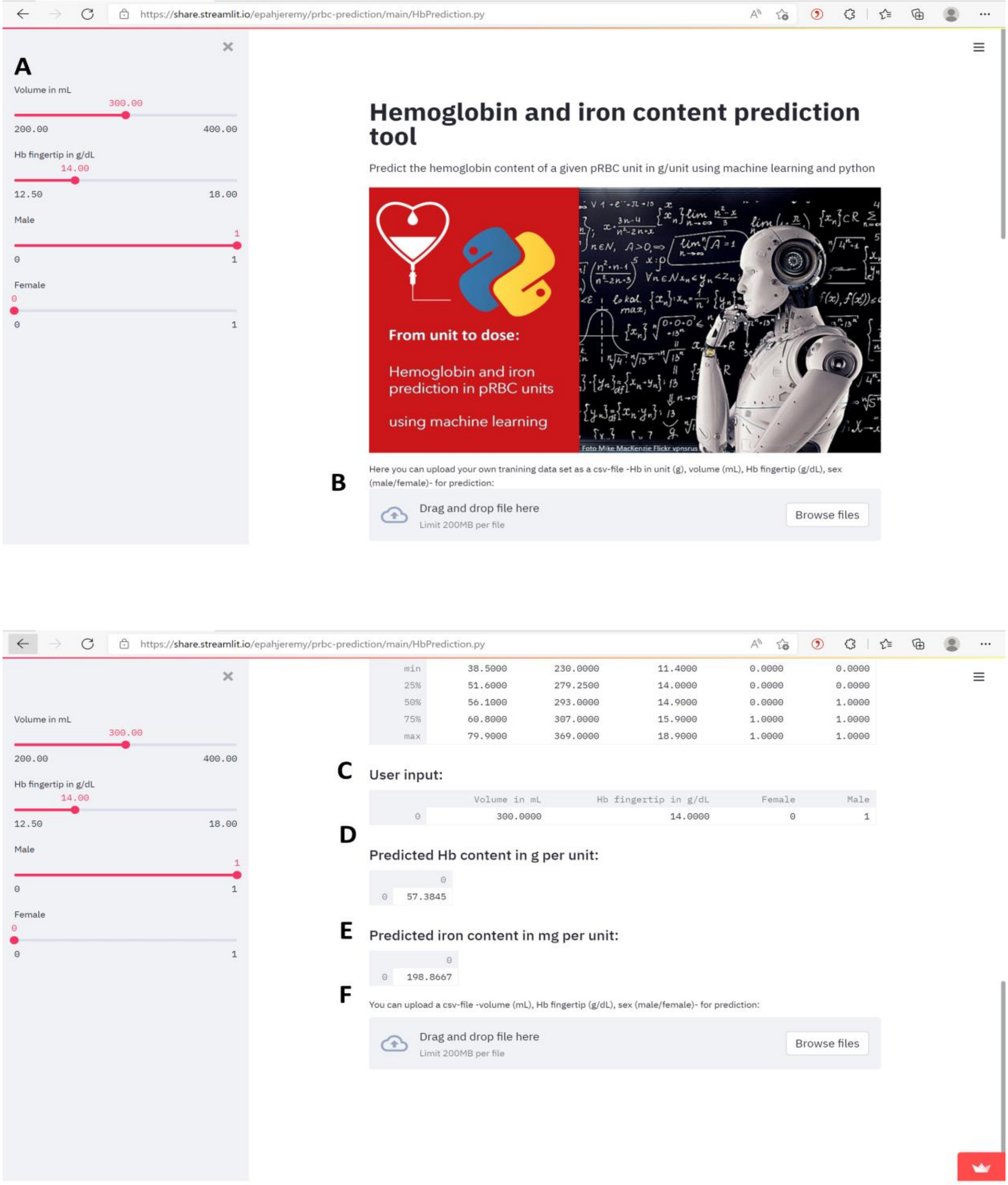

Figure S8 Web application for hemoglobin and iron content prediction (https://epahjeremy-prbc-prediction-hbprediction-dceyew.streamlitapp.com)

A: Input slider for prediction of individual pRBC unit

B: Option to upload customized training data set

C: Display for user input from A

D: Prediction result for Hb in g per unit

E: Prediction result for iron in mg per unit

F: Option to upload a data set containing pRBC units for prediction.

Figure S9

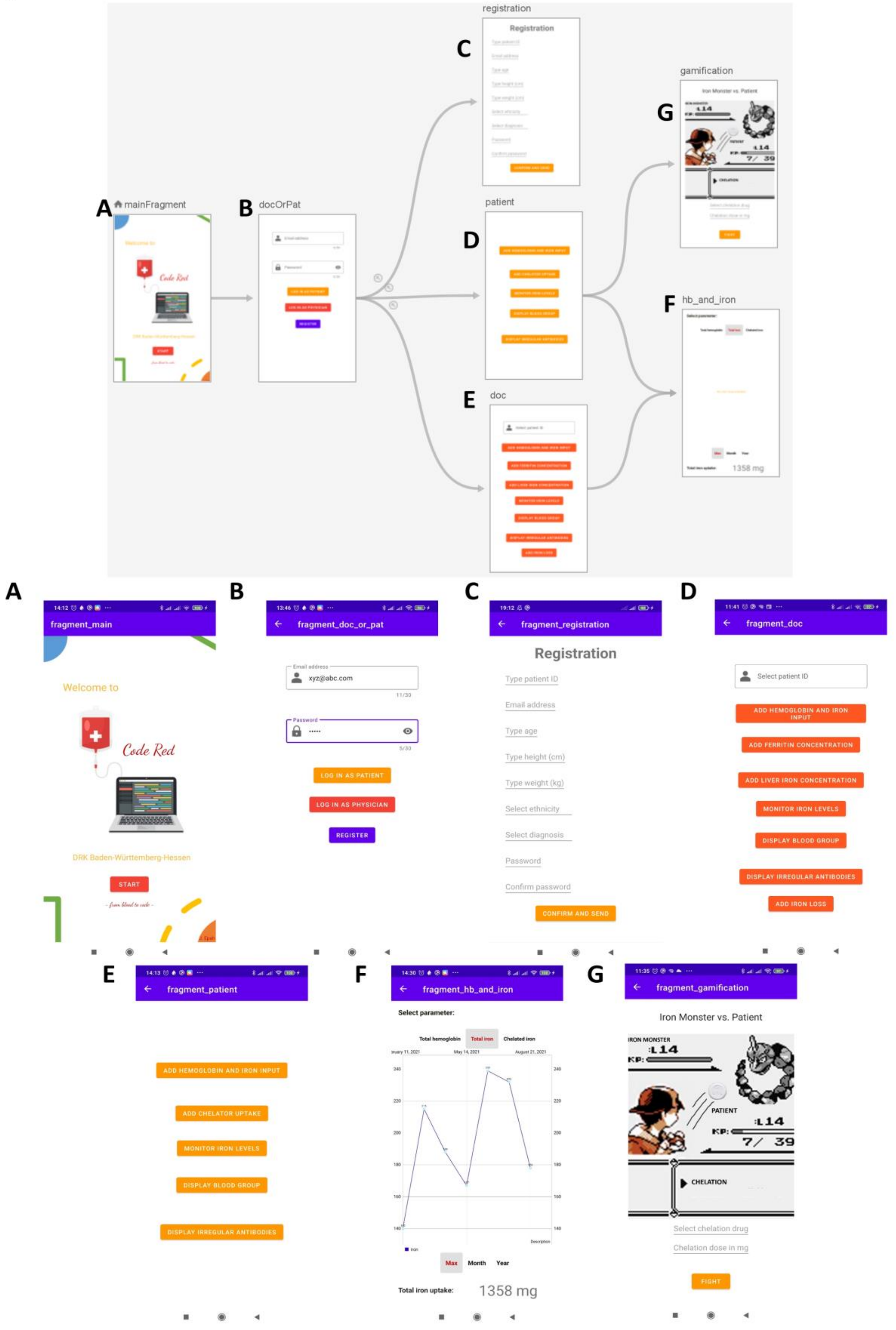

Figure S9 Navigation graph of a proposed concept for a mobile application

A: Start view

B: Log in view

C: Registration view

D: Patient view

E: Physician view

F: Graphical monitoring of iron levels

G: Possible gamification option for pediatric patients.

References for the Supporting Information

[1] O. Arslan, S. Toprak, M. Arat, Y. Kayalak, *Transfusion* **2004**, *44*, 485.

[2] E. Atilla, S.K. Toprak, S. Civriz Bozdağ, P. Topçuoğlu, Ö. Arslan, *Turkish journal of haematology official journal of Turkish Society of Haematology* **2017**, *34*, 244.

[3] N. Agnihotri, L. Pal, M. Thakur, P. Kumar, *Blood transfusion = Trasfusione del sangue* **2014**, *12*, 520.

[4] A.F. List, M.R. Baer, D.P. Steensma, A. Raza, J. Esposito, N. Martinez-Lopez, C. Paley, J. Feigert, E. Besa, *Journal of clinical oncology official journal of the American Society of Clinical Oncology* **2012**, *30*, 2134.

[5] P.L. Carver, Ed., *Essential Metals in Medicine: Therapeutic Use and Toxicity of Metal Ions in the Clinic*, De Gruyter **2019**.
